# Position Tracking Techniques Using Multiple Receivers for Anti-Drone Systems

**DOI:** 10.3390/s21010035

**Published:** 2020-12-23

**Authors:** Jae-Min Shin, Yu-Sin Kim, Tae-Won Ban, Suna Choi, Kyu-Min Kang, Jong-Yeol Ryu

**Affiliations:** 1 Department of Information and Communications Engineering, Gyeongsang National University, Tongyeong 53064, Korea; tiswoars1995@gnu.ac.kr (J.-M.S.); 2019211010@gnu.ac.kr (Y.-S.K.); twban35@gnu.ac.kr (T.-W.B.); 2 Electronics and Telecommunications Research Institute (ETRI), Daejon 34129, Korea; sunachoi@etri.re.kr (S.C.); kmkang@etri.re.kr (K.-M.K.)

**Keywords:** drone position tracking, multiple Bluetooth receivers, tracking algorithm

## Abstract

The need for drone traffic control management has emerged as the demand for drones increased. Particularly, in order to control unauthorized drones, the systems to detect and track drones have to be developed. In this paper, we propose the drone position tracking system using multiple Bluetooth low energy (BLE) receivers. The proposed system first estimates the target’s location, which consists of the distance and angle, while using the received signal strength indication (RSSI) signals at four BLE receivers and gradually tracks the target based on the estimated distance and angle. We propose two tracking algorithms, depending on the estimation method and also apply the memory process, improving the tracking performance by using stored previous movement information. We evaluate the proposed system’s performance in terms of the average number of movements that are required to track and the tracking success rate.

## 1. Introduction

Recently, drones have developed from the existing radio frequency (RF) signal control method to the combination of communication networks, such as LTE and 5G, thereby increasing the controllable distance and reducing the response time. Furthermore, the development of battery technology has increased flight time and, as satellite positioning technology has been developed, the accuracy of the positioning has been improved. Because of the development of drone technologies, the field of drones is increasingly diversified, and the drone market continues to grow [1,2,3]. However, the diversity of using drones can carry out the abuse of drone technologies, such as crime or terrorism. As the demand for drones increases, the use of an unauthorized drone is also increasing. Unauthorized drones can cause accidents that collide with flying objects, damage property, or injure people for malicious purposes. Somebody can also use unauthorized drones in order to transport illegal objects and invade privacy, such as unlawful filming. Recently, with Saudi Arabia’s oil facilities under the threat of terrorism by drones, the need for investment and research into anti-drone technologies has emerged worldwide [4,5,6,7,8,9,10,11,12]. Anti-drone stands for a system that detects, identifies, tracks, and incapacitates illegal drones. The anti-drone market is expected to grow from USD 499 million in 2018 to USD 2.27 billion in 2024 [3]. The technique for the detection and tracking of unauthorized drones is the core technology in the anti-drone system [13,14].

The most widely used methods in drone detection are the techniques while using radar, radio frequency (RF) signals, and images [13]. A typical drone detection method is a radar that detects the location of illegal drones by reflected radio waves [4]. However, radar is expensive to install and maintain, and it is hard to detect small-size drones. As a radar replacement technology to solve this problem, various methods were considered, such as using the physical characteristics of a RF signal that is used for drone control distinguished from a mobile device [5], and monitoring drones by camera images [6,7,8], and detecting an acoustic signal that is generated by a drone [9]. However, the method using the physical characteristics of the RF signal can be hard to detect illegal drones if drones use RF signals that resemble the RF characteristics of other mobile devices. In addition, the method of using the camera can be expensive, due to the high processing requirements for processing a large amount of image data, and the detection speed is relatively low. The acoustic array method can be hard to detect the drone’s acoustic signal, due to the self-interference. Furthermore, the method that is presented above has difficulty in distinguishing illegal drones hidden in authorized drone groups. The drone authentication system for identifying unauthorized drones has been actively investigated with the drone traffic management system [11,12]. Therefore, in this paper, we assume that drones transmit authentication information through RF signals aad consider RF-based detection that tracks the RF signal of the unauthenticated drone among the drone’s detected RF signals. RF signal-based anti-drone system while using the RF control signal that was exchanged between the drone, and its remote controller has already been studied [10]. However, we propose an aerial solution while using a tracking drone, not the stationary solution above. Aerial solutions approach targets, so you can expect higher accuracy and reduce the threat to legitimate communication systems [8].

In the anti-drone system, the core technology is the position tracking technology in order to detect and track unauthorized drones’ exact location. The positioning system has been steadily investigated, as interest in location-based services (LBS) has increased due to advances in IoT and mobile device technologies [15,16,17]. Conventional positioning systems have been developed based on the GPS signal. However, the GPS based positioning system cannot be directly applied to the drone position tracking system, because it is hard to find GPS information from the unauthorized drones at the mobile tracker. Therefore, instead of the GPS based techniques, we consider the tracking that is based on Wi-Fi and Bluetooth low energy (BLE) technologies in a Wireless Personal Area Network (WPAN) [16,17]. Based on BLE technology development, the market of LBS using BLE has grown rapidly [18,19], and it application has been attempted in various environments, such as airports [20] and museums [21]. Furthermore, the battery life has been dramatically extended, and it makes it suitable for long-term monitoring and surveillance and easy to mount on drones at low prices and small. Thus, we consider the drone position tracking system that is based on BLE technology [18,19,20,21,22,23].

Research on existing BLE-based location tracking techniques is based on a fingerprinting approach while using the received signal strength indication (RSSI) database [23,24,25], tracking an object’s position while using the trilateration method, and the RSSI-distance conversion formula using a propagation model [26,27,28,29,30]. In [16,24,31], the authors proposed location tracking techniques that combine the existing methods with various sensors to improve the performance. Fingerprinting based location tracking requires building a database in advance and investing time in maintenance. In particular, the fingerprinting cannot be applied to the tracking method using the autonomous flight drone, because the receiver moves dynamically, and the environment changes constantly and frequently. In addition, the method to enhance the precision of location tracking in combination with various sensors, such as gyro, acceleration, and inertial sensors, cannot be applied in the anti-drone system, because the target is the unauthorized drone that hides its identity. Thus, it is hard to receive sensor information from the target. Therefore, we apply the distance conversion formula while using the propagation model to the position tracking in the anti-drone system. However, the method that simply uses the propagation model has inferior positioning accuracy due to reflection and interference [16]. In order to improve the accuracy, the refining algorithms that combine the calibration of RSSI coefficients, iterative trilateration, and smoothing algorithm to minimize the dynamic signal fluctuation were proposed in [27]. In [28], a method of using frequency diversity and spatial diversity to suppress RSSI fluctuation was proposed, and a Kalman filter was applied in order to improve the accuracy of trilateration [29,30]. However, the previous positioning algorithms directly estimate the target’s location based on RSSI and, hence, the performance seriously depends on the accuracy of RSSI. Furthermore, the algorithm has to consume a large amount of time to increase RSSI’s accuracy, thus decreasing the tracking speed. Therefore, we propose the position tracking algorithms that gradually approach the target and are robust to the RSSI accuracy and estimation error of the position.

In this paper, we propose the position tracking techniques while using multiple BLE receivers. In contrast to the existing algorithms, the tracker gradually tracks the target that is based on the estimated location that was obtained from RSSI values at multiple receivers. In the proposed algorithms, the tracker first determines the distance and angle for movement using RSSI values at receivers. Subsequently, it gradually moves toward the target according to the moving distance and angle. We propose two tracking algorithms, the constant distance and quantized angle constant distance and quantized angle (CDQA) algorithm and the adaptive distance and continues angle (adaptive distance and continues angle (ADCA)) algorithm, according to the method for determining the moving distance and angle. In order to reduce the effect of the estimation error that is caused by RSSI’s inaccuracy, we apply the memory process, which exploits the previous movement information, in the proposed algorithms.

The rest of the paper is organized, as follows. In Section 2, we introduce a location tracking system while using multiple BLE receivers. In Section 3, we propose position tracking algorithms and a method for improving the tracking algorithms’ performance. Section 4 evaluates the performance of the position tracking algorithms proposed in this paper through simulations and compares the performance through numerical results. Finally, we will present conclusions in Section 5.

## 2. System Model

**Figure 1:**
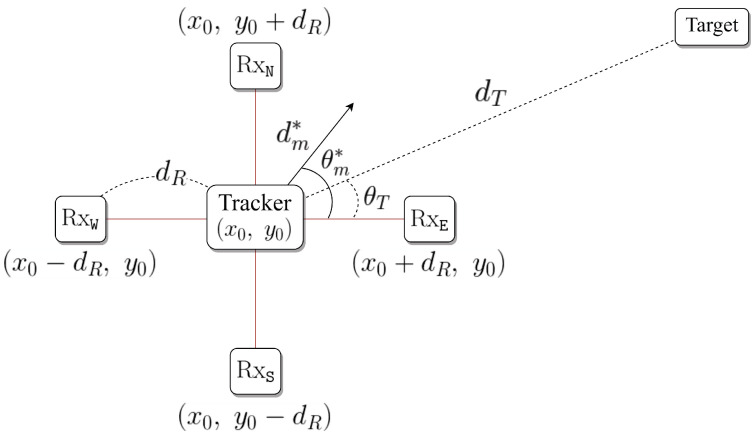
Tracking system model.

We consider a tracking system where the tracker equips four Bluetooth receivers, as shown in Figure 1. The tracker equips four receivers, and the physical distance between the tracker’s center and each receiver is $d_{R}$. The receivers are located on the vertical and horizontal axes with $d_{R}$. Because the tracker equips the fixed receivers, the distance between the tracker’s center and each receiver, $d_{R}$, is unchanged at each movement. In our tracking system, because the tracker does not know an initial location of the target before receiving RSSI, the receiver has to listen RSSI in all directions. Therefore, we assume that the radiation pattern is isotropic, and the antenna gain is the same. We define an initial location of the tracker as $\left(x_{0},\;y_{0}\right)$ and, thus, the locations of four receivers are given by $\left(x_{0},\;y_{0}+d_{R}\right)$, $\left(x_{0}+d_{R},\;y_{0}\right)$, $\left(x_{0},\;y_{0}-d_{R}\right)$, and $\left(x_{0}-d_{R},\;y_{0}\right)$, respectively. The tracking space for the drones is a three-dimensional space. However, in the indoor environment, the drones cannot fly at a high altitude. Thus, the altitude difference between the tracker and target is relatively small when compared to the distance between them. Therefore, we simplify the 3-dimensional tracking space to 2-dimensional tracking space. The target is located apart from the tracker the distance $d_{T}$ and the angle $\theta_{T}$, which is an angle between the horizontal axis and target. The tracker does not know $d_{T}$ and $\theta_{T}$ before the completion of the tracking.

The tracker traces the target based on the RSSI values that were obtained from the target’s broadcasting signal at the receivers. In our tracking system, we assume that the target does not move during the tracking and thus, if the tracker cannot approach the target in a certain time, the tracking is failed. Because the tracker and target do not move during the collection for RSSI, we use a static channel model for RSSI. We assume the RSSI follows the Indoor Log-Distance path loss model [32], as

(1) $$\begin{array}{c} RSSI=RSSI_{d_{0}}-10n\log\left(\frac{d}{d_{0}}\right)+X_{\sigma }, \end{array}$$

where *n* is the path loss exponent and $X_{\sigma }$ is a normal random variable in dB having a standard deviation of σ dB. The path loss at the reference distance $d_{0}(=1$ m) is defined by $RSSI_{d_{0}}$. At each movement, the tracker collects the RSSI values during the collection period and then calculates the average values of RSSI to track the target’s location. During the RSSI collection period, the tracker does not move and collect RSSI. The cycle of receiving the RSSI signal is defined by *T* and the number of received RSSI during the collection period is given by *w*. Thus, the total collection period is given by w·T. Based on the average RSSI values at receivers, the tracker determines the moving distance $d_{m}^{*}$ and angle $\theta_{m}^{*}$ and moves by the distance $d_{m}^{*}$ in the direction $\theta_{m}^{*}$. The moving distance and angles are determined according to the tracking algorithms, which will be presented in Section 3. The number of movement to track the target is defined by $n_{m}$ and it is bounded by the maximum number of the movement $n_{\max}$. Thus, if $n_{m}>n_{\max}$, then the tracking is failed. Otherwise, for the case of $n_{m}\leq n_{\max}$, when the tracker approaches within the threshold distance $d_{\mathrm{th}}$ from the target, i.e., $d_{T}\leq d_{\mathrm{th}}$, the tracking is successfully completed.

In the tracking system, it is important both how to fast track the target and how to successfully track the target. Therefore, as the performance measure, we provide the success rate that is based average tracking time, which considers both the success rate of the tracking and average tracking time for the success tracking event. The success rate based average tracking time is defined by the average tracking time divided by the success rate of the tracking as

(2) $$\begin{array}{c} t_{s}=\frac{t_{s,avg}}{R_{suc}}. \end{array}$$

For the case that the tracker successfully tracks the target *k* times in the total *K* attempts for the tracking, the success rate based average tracking time can be represented by

(3) $$\begin{array}{cc} t_{s}\; & =\frac{\frac{1}{k}\times \sum_{i=1}^{k}\left(n_{m}\left(i\right)\times w\times T+d_{M}\left(i\right)/v\right)}{\frac{k}{K},}\\ \; & =\frac{K\times \sum_{i=1}^{k}\left(n_{m}\left(i\right)\times w\times T+d_{M}\left(i\right)/v\right)}{k^{2}}, \end{array}$$

where $n_{m}\left(i\right)$ is the number of the movement for the completion of the *i*-th tracking event and $d_{M}\left(i\right)$ is a summation of the total movement distance of tracker at the *i*-th tracking event. The movement speed of the tracker is defined by *v*.

**Figure 2:**
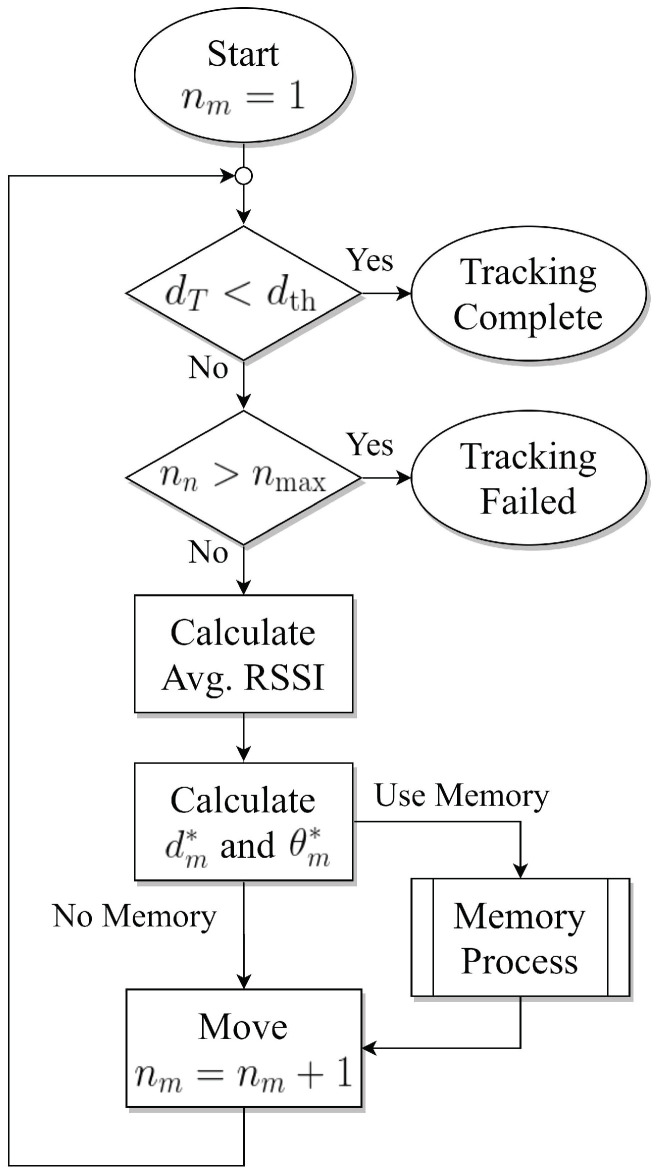
Tracking Process.

In addition, we apply a memory process for the tracking algorithms, which stores the information of the previous movement steps and reduces the probability to move to the wrong direction that is based on the stored information. Figure 2 presents the overall tracking process. In details of using the memory and the algorithms to determine the moving distance and moving angles are proposed in the following section.

## 3. Tracking Algorithms

In this section, we propose the tracking algorithms that trace the target that is based on the moving distance and angle. Furthermore, we apply a simple memory process to improve the tracking algorithms by using previous movement information.

### 3.1. CDQA Tracking Algorithm

We first propose a simple CDQA based tracking algorithm. At each movement step, the tracker moves a predefined distance, which is a constant value, toward the direction, which is selected from the predefined four directions with a 90 degree angle difference from each other. In the CDQA tracking algorithm, the moving distance and angle are defined as

(4) $$\begin{array}{cc} d_{m} & =D_{m}, \end{array}$$

(5) $$\begin{array}{cc} \theta_{m} & \in \left\{\theta_{E}=0,\;\theta_{N}=\frac{\pi }{2},\;\theta_{W}=\pi ,\;\theta_{S}=\frac{3}{2}\pi \right\}, \end{array}$$

where $D_{m}$ is the predefined constant value. By comparing the average RSSI values at the receivers, the tracker selects the moving angle $\theta_{m}$ as the direction of the receiver that has the maximum average RSSI value, as

(6) $$\begin{array}{c} \theta_{m}^{*}=\underset{\theta_{m}\in \left\{\theta_{E},\theta_{N},\theta_{W},\theta_{S}\right\}}{\arg\;\max}\;{\bar{RSSI}}_{i}, \end{array}$$

where $\bar{RSSI_{i}}\in \left\{{\bar{RSSI}}_{E},{\bar{RSSI}}_{N},{\bar{RSSI}}_{W},{\bar{RSSI}}_{S}\right\}$ is the average RSSI value at each receiver.

In the CDQA tracking algorithm, therefore, the moving location of the tracker is determined by

(7) $$\left(x,\;y\right)=\left(D_{m}\cos\theta_{m}^{*},\;D_{m}\sin\theta_{m}^{*}\right).$$

### 3.2. ADCA Tracking Algorithm

We propose an ADCA based tracking algorithm in order to track the target efficiently. In a ADCA algorithm, the tracker calculates the moving distance and angle by using the average RSSI values at the receivers at each movement step.

By using (1) and the average RSSI value that was obtained from each receiver, the tracker first estimates the distance between the target and each receiver as

(8) $$\begin{array}{c} {\tilde{d}}_{i}=10^{\frac{-{\bar{RSSI}}_{i}+RSSI_{d_{0}}}{10\cdot n}},\;i\in \left\{E,\;N,\;W,\;S\right\}. \end{array}$$

There exists an estimation error of the estimated distance, due to the random variable $X_{\sigma }$ and the amount of the error can be determined by the number of received RSSI, *w*, during the collection period. If *w* is sufficiently large, then the distance that is calculated from the average RSSI value approaches the actual distance.

Subsequently, the tracker selects the nearest vertical and horizontal distances, ${\tilde{d}}_{v}$ and ${\tilde{d}}_{h}$, from the estimated distances as

(9) $$\begin{array}{cc} {\tilde{d}}_{v} & =\min\left({\tilde{d}}_{N},\;{\tilde{d}}_{S}\right), \end{array}$$

(10) $$\begin{array}{cc} {\tilde{d}}_{h} & =\min\left({\tilde{d}}_{E},\;{\tilde{d}}_{W}\right). \end{array}$$

Based on ${\tilde{d}}_{v}$ and ${\tilde{d}}_{h}$, the tracker determines the quadrant to move in the current movement step.

**Figure 3:**
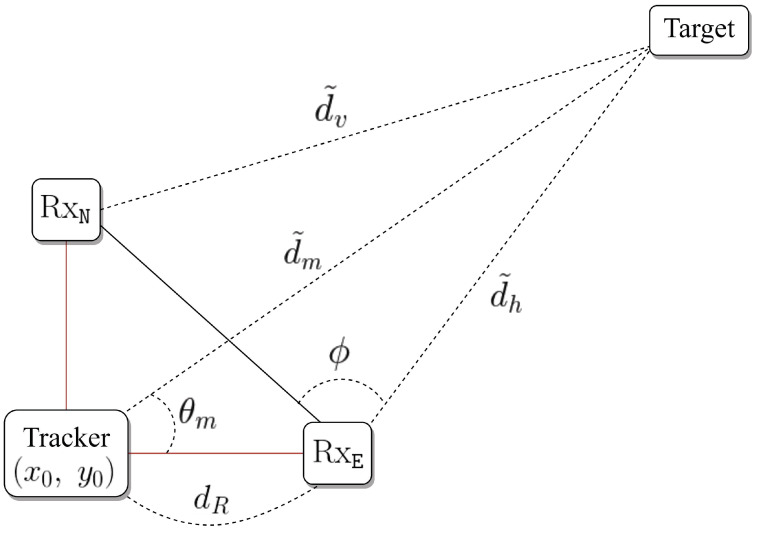
Example in the first quadrant.

As an example of the estimation of $d_{m}^{*}$ and $\theta_{m}^{*}$, the configuration where the target is located in the first quadrant is given in Figure 3. By using the law of cosines, the distance between the tracker and target can be estimated as

(11) $$\begin{array}{c} {\tilde{d}}_{m}=\sqrt{{\left({\tilde{d}}_{h}\sin\left(\phi +\frac{\pi }{4}\right)\right)}^{2}+{\left(d_{R}-{\tilde{d}}_{h}\cos\left(\phi +\frac{\pi }{4}\right)\right)}^{2}}, \end{array}$$

where the angle ϕ is given by

(12) $$\begin{array}{c} \phi =\arccos\left(\frac{2d_{R}^{2}+{\tilde{d}}_{h}^{2}-{\tilde{d}}_{v}^{2}}{2\sqrt{2}d_{R}\cdot {\tilde{d}}_{h}}\right). \end{array}$$

For the case that the target is in the first quadrant, by using the estimated distances and law of cosines, the moving angle is determined as

(13) $$\begin{array}{c} \theta_{m}=\arccos\left(\frac{d_{R}^{2}+{\tilde{d}}_{m}^{2}-{\tilde{d}}_{h}^{2}}{2d_{R}\times {\tilde{d}}_{m}}\right). \end{array}$$

For the general case, according to the quadrant for the movement, the moving angle $\theta_{m}^{*}$ of the tracker is determined by

(14) $$\begin{array}{c} \theta_{m}^{*}=\left\{\begin{array}{cc} \theta_{m}, & {\tilde{d}}_{h}={\tilde{d}}_{E},\;{\tilde{d}}_{v}={\tilde{d}}_{N}\\ \pi -\theta_{m}, & {\tilde{d}}_{h}={\tilde{d}}_{W},\;{\tilde{d}}_{v}={\tilde{d}}_{N}\\ \pi +\theta_{m}, & {\tilde{d}}_{h}={\tilde{d}}_{W},\;{\tilde{d}}_{v}={\tilde{d}}_{S}\\ 2\pi -\theta_{m}, & {\tilde{d}}_{h}={\tilde{d}}_{E},\;{\tilde{d}}_{v}={\tilde{d}}_{S} \end{array}\right.. \end{array}$$

However, there exists the case that the condition of a triangle cannot meet, due to the estimation error. In this case, we define a proportional formula relative to the selected vertical and horizontal distance as

(15) $$\begin{array}{c} {\tilde{d}}_{h}:{\tilde{d}}_{v}=\theta_{m}:\frac{\pi }{2}-\theta_{m}, \end{array}$$

and, from the proportional formula, we can obtain $\theta_{m}$ as

(16) $$\begin{array}{c} \theta_{m}=\frac{\pi \times {\tilde{d}}_{h}}{2\left({\tilde{d}}_{v}+{\tilde{d}}_{h}\right)}. \end{array}$$

By substituting (16) into (13), we also obtain the following quadratic equation

(17) $$\begin{array}{c} {\tilde{d}}_{m}^{2}-2\cos\theta_{m}\times d_{R}\times {\tilde{d}}_{m}+d_{R}^{2}-{\tilde{d}}_{h}^{2}=0, \end{array}$$

and, according to the quadratic formula, ${\tilde{d}}_{m}$ can be obtained as

(18) $$\begin{array}{cc} {\tilde{d}}_{m} & =d_{R}\cos\theta_{m}\pm \sqrt{{\left(d_{R}\cos\theta_{m}\right)}^{2}-d_{R}^{2}+{\tilde{d}}_{h}^{2}} \end{array}$$

(19) $$\begin{array}{cc} \; & =d_{R}\cos\theta_{m}\pm \sqrt{D}, \end{array}$$

where $D={\left(d_{R}\cos\theta_{m}\right)}^{2}-d_{R}^{2}+{\tilde{d}}_{h}^{2}$. In the tracking system, if the tracker approaches to the target within $d_{R}\left(<d_{\mathrm{th}}\right)$, the tracking is already completed. Thus, the estimated distance in (18) can be rewritten by

(20) $$\begin{array}{cc} {\tilde{d}}_{m}\; & =d_{R}\cos\theta_{m}+\sqrt{{\left[D\right]}^{+}}, \end{array}$$

where the operation ${\left[\cdot \right]}^{+}$ is defined by ${\left[x\right]}^{+}=\max\left(0,x\right)$.

Therefore, if the condition of the triangle cannot meet due to the estimation error, then ${\tilde{d}}_{m}$ and $\theta_{m}$ are obtained by (20) and (16). Otherwise, ${\tilde{d}}_{m}$ and $\theta_{m}$ are obtained by (11) and (13).

In addition, due to the estimation error, the difference between the estimated distance and the actual distance can be large, and results in an unnecessary long detour, which reduce the performance. Therefore, for the moving distance, we normalize the estimated distance and restrict the maximum moving distance as

(21) $$\begin{array}{c} d_{m}^{*}=\min\left(\frac{{\tilde{d}}_{m}}{\alpha },\;d_{m}^{\max}\right), \end{array}$$

where α(≥1) and $d_{m}^{\max}$ are the constant values.

In the ADCA tracking algorithm, the moving location of the tracker is determined by

(22) $$\begin{array}{c} \left(x,\;y\right)=\left(d_{m}^{*}\cos\theta_{m}^{*},\;d_{m}^{*}\sin\theta_{m}^{*}\right), \end{array}$$

where $d_{m}^{*}$ and $\theta_{m}^{*}$ are given in (21) and (14), respectively.

### 3.3. Algorithm with Memory Process

When the target is located far from the tracker, the tracker can move in the wrong direction due to the distance and angle estimation error. Once the tracker moved in the wrong direction, the probability of occurring the estimation error increases in the next movement step, because the distance between the tracker and target becomes longer. Consequently, an error propagation of the estimation can occur, which decreases the probability of a successful completion of the tracking.

**Figure 4:**
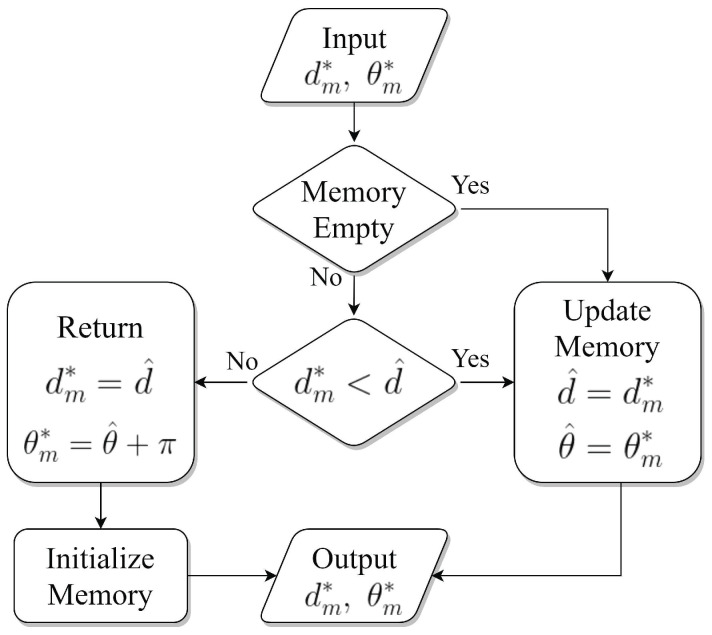
Memory Process.

We apply the memory process to the proposed algorithms in order to resolve the estimation error propagation. The tracker stores the information of the previous movement and location in the memory. At each movement step, the tracker calculates the estimated distance at the current step and compares it with that of the previous step. Subsequently, suppose that the estimated distance becomes longer compared to that of the previous step. In this case, the tracker returns to the previous step’s location and, again, receives the RSSI signals from the target. Otherwise, the tracker updates the memory by storing the information on the location of the current step. In the success rate based average tracking time, the number of the return is included in the number of the movement, moving distance, and time. Figure 4 provides the flowchart of the memory process.

### 3.4. Trilateration Based Tracking Algorithm

We introduce the trilateration based tracking algorithm in order to compare with the proposed tracking algorithms [24,27,29,30,31]. By applying the trilateration method to our tracking environment, the trilateration formula to estimate the location of the target is given by

(23) $$\begin{array}{c} {\left(x-d_{R}\right)}^{2}+y^{2}={\tilde{d}}_{E}^{2}, \end{array}$$

(24) $$\begin{array}{c} x^{2}+{\left(y-d_{R}\right)}^{2}={\tilde{d}}_{N}^{2}, \end{array}$$

(25) $$\begin{array}{c} {\left(x+d_{R}\right)}^{2}+y^{2}={\tilde{d}}_{W}^{2}, \end{array}$$

(26) $$\begin{array}{c} x^{2}+{\left(y+d_{R}\right)}^{2}={\tilde{d}}_{S}^{2}. \end{array}$$

The tracker first estimates the distance between the target and receivers by (8). Subsequently, the tracker estimates the location of the target by calculating the solution of (23)–(26) as

(27) $$\begin{array}{c} \left(x,\;y\right)=\left(\frac{{\tilde{d}}_{W}^{2}-{\tilde{d}}_{E}^{2}}{4d_{R}},\;\frac{{\tilde{d}}_{S}^{2}-{\tilde{d}}_{N}^{2}}{4d_{R}}\right). \end{array}$$

The trilateration based tracking algorithm can also apply the memory process to improve the performance.

## 4. Simulation Results

In this section, we evaluate the proposed algorithms’ performance in terms of $t_{s}$, the average tracking time based on the success rate given in (3). The RSSI signal for estimating distances is generated by (1), the path loss exponent is n=3, and the random variable $X_{\sigma }$ has σ=7 dB. The simulation parameters that are commonly used in the figures are given the following: the distance between the tracker and receiver is $d_{R}=0.5$ m, the maximum number of the movement $n_{\max}=500$, the cycle of receiving RSSI is T=0.1 s, the number of received RSSI for averaging is w=10, the movement speed of the tracker is v=5 m/s, and the threshold distance is $d_{\mathrm{th}}=1$ m. By considering the coverage and reception stability of RSSI, we set the distance between the tracker and target as $d_{T}\leq 30$ m. The path loss at the reference distance $RSSI_{d_{0}}=-50$ dBm. Although the target can move, this paper assumes a scenario of tracking a fixed target. Additionally, we simulate 10,000 times for each scenario to obtain the simulation results by averaging.

**Figure 5:**
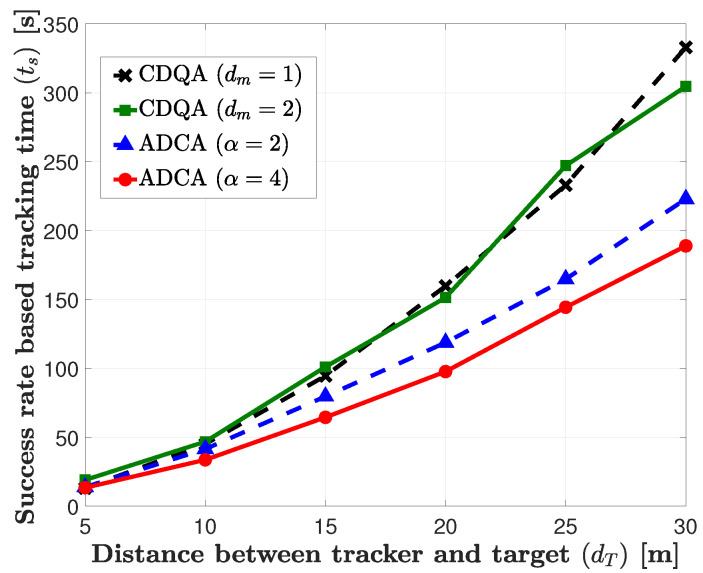
Performance Comparison of constant distance and quantized angle (CDQA) and adaptive distance and continues angle (ADCA).

In Figure 5, we plot the average tracking time that is based on the success rate, $t_{s}$, of the proposed tracking algorithms according to the distance between the tracker and the target, $d_{T}$. In this figure, we can observe that the ADCA based tracking algorithm outperforms the CDQA based tracking algorithm for all $d_{T}$, because the ADCA algorithm has greater freedom to move to the target in terms of the distance and angle. For the CDQA algorithm, when the distance between the tracker and target is short, e.g., $d_{T}<15$ m, the algorithm with the short moving distance ($D_{m}=1$ m) outperforms the algorithm with a long moving distance ($D_{m}=2$ m). In this case, it is beneficial that the tracker moves elaborately to find the target. However, when the target is located far from the tracker, e.g., $d_{T}>25$ m, the algorithm with $D_{m}=2$ m yields better performance than with $D_{m}=1$ m. In this case, it is beneficial to approach the target with a long moving distance rapidly.

For the ADCA algorithm, the algorithm with α=4 outperforms that with α=2. Because the moving distance with α=2 is twice longer than that with α=4, the tracker has to conservatively approach the target in order to reduce the effect of moving in the wrong direction in the ADCA algorithm.

**Figure 6:**
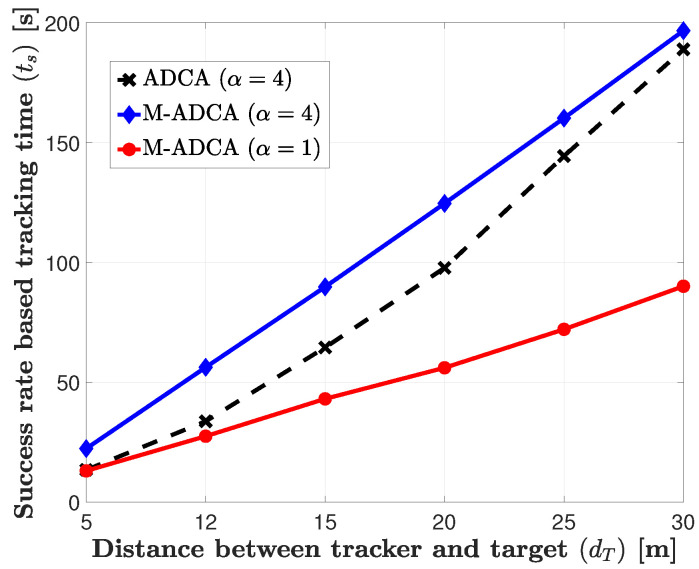
Performance Comparison of ADCA and M-ADCA (ADCA with Memory).

Figure 6 shows the performance improvement by applying the memory process in the ADCA tracking algorithm. In the ADCA algorithm, we take a heuristic conservative approach, such as a normalize factor α(≥1), because it has significantly reduced performance by moving in the wrong direction, as shown in Figure 5. By applying the memory process, however, the tracker can go back to the previous location when it moves in the wrong direction. Thus, the tracker does not have to conservatively move to the target, i.e., α=1. By comparing the ADCA with α=4 and the memory applied ADCA (M-ADCA) with α=1, we can observe that the M-ADCA outperforms the ADCA for all distances, and the performance gap becomes larger as the distance increases. Therefore, the performance of the tracking system can be significantly improved by applying the memory process.

**Figure 7:**
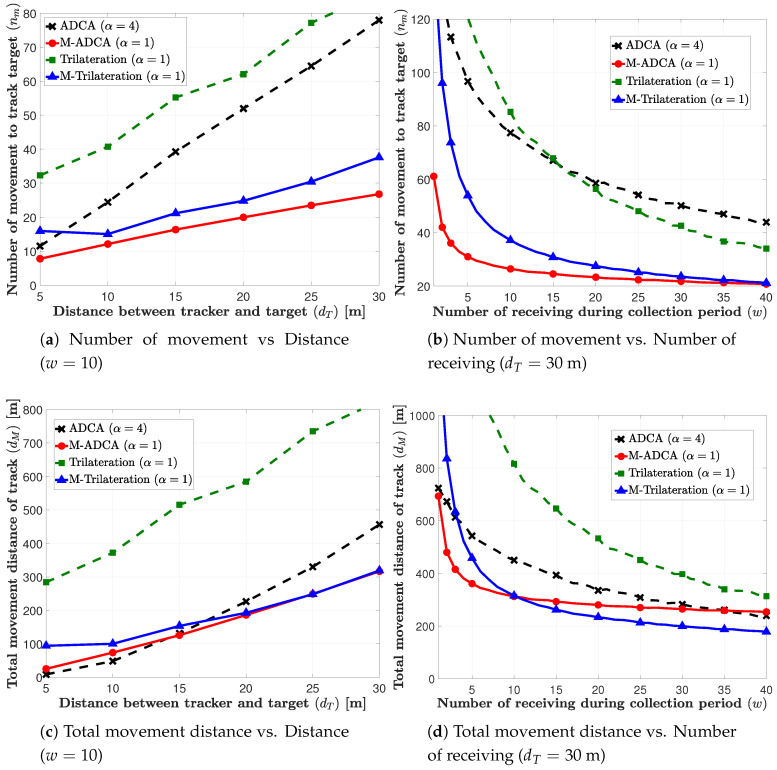

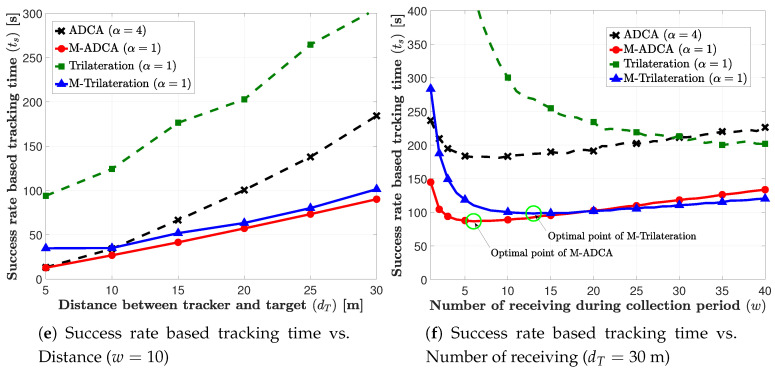
Comparison of performance of the proposed tracking algorithms and trilateration algorithm. ($d_{m}^{\max}$ = 10).

In Figure 7, we compare the proposed tracking algorithms and the existing trilateration based algorithm and memory applied trilateration (M-Trilateration) algorithm in terms of the number of movement, total moving distance, and the success rate that is based on the average tracking time. In Figure 7a,b, we plot the number of movements for tracking, $n_{m}$, the distance between the tracker and the target, $d_{T}$, and the number of that were received RSSIs during the collection period, *w*, respectively. In these figures, we first observe the memory process improves the performance of the trilateration-based algorithm as well as the proposed algorithm. Figure 7a shows that the proposed tracking algorithms, ADCA and M-ADCA, outperform the conventional trilateration based algorithms, Trilateration and M-Trilateration, respectively, for all $d_{T}$. In Figure 7b, we observe that the proposed algorithms’ performance outperform the trilateration based algorithms for small and medium *w*. However, the trilateration based algorithms approach to or outperform the proposed algorithms for large *w*. When *w* is large, the estimation error can be reduced and, thus, the conventional algorithms, which are sensitive to the estimation error, yield good performance. However, the system with large *w* consumes a long time to receive the RSSIs and, thus, the overall tracking performance can be worse, due to the increased tracking time. In Figure 7c,d, the total movement distances for tracking, $d_{M}$, are plotted according to $d_{T}$ and *w*, respectively. In these figures, we can observe the similar results with Figure 7a,b. The proposed algorithms outperform the conventional algorithms for all $d_{T}$ and the M-Trilateration algorithm yields better performance than M-ADCA for large *w*, in terms of the total movement distance.

In Figure 7e,f, we evaluate the overall performance of the proposed and conventional algorithms in terms of the success rate based tracking time, $t_{s}$, which considers both tracking accuracy and tracking speed. In Figure 7e, by comparing the ADCA and M-ADCA, we first observe that the M-ADCA algorithm obtains a maximum gain of approximately 51% ($d_{T}=30$ m), and the average gain about 34% by applying memory process. The proposed ADCA algorithm as compared to the conventional Trilateration obtains a maximum gain of about 86% ($d_{T}=5$ m) and an overall average gain of about 60% for all distances. When comparing to the conventional M-Trilateration algorithm, the proposed M-ADCA algorithm can achieve a maximum gain of about 63% ($d_{T}=5$ m) and an overall average gain of about 23% for all distances. In Figure 7f, we can observe that there exists the optimal value of *w* that minimizes $t_{s}$. For small *w*, the algorithms’ success rate based tracking times decrease as *w* increases, because the increased *w* reduces the estimation error. However, for large *w*, the algorithms’ performance worsens as *w* increases, due to the increased total tracking time. Because *w* is a system design parameter, we can optimize *w* to minimize $t_{s}$. Hence, we can evaluate the performance of the algorithms by comparing the optimal points of $t_{s}$, which are marked by green circles in Figure 7f. Consequently, by comparing the optimal $t_{s}$, the proposed M-ADCA algorithm obtains 11.8% gain as compared to the conventional M-Trilateration algorithm for $d_{T}=30$ m.

## 5. Conclusion and Discussion

In this paper, we proposed the position tracking system with multiple Bluetooth receivers. In the proposed tracking system, the tracker estimated the target’s location while using the average RSSI at each receiver and then approached the target sequentially based on the estimated distance and angle. Additionally, we proposed the tracking algorithms. The CDQA algorithm selects the constant distance and quantized angle for movement to the target, and the ADCA algorithm calculates the moving distance and angle that are proportional to the received average RSSI values. Furthermore, we applied the memory process that reduces the effect of the estimation error using the movement’s previous information. In the simulation results, by comparing the conventional tracking algorithm that is based on trilateration, we showed that our proposed algorithm outperforms the traditional algorithm by a maximum of approximately 86% and we obtain a maximum gain of about 51% by applying the memory process.

In this paper, we proposed the tracking algorithms while using mathematical models and evaluated the theoretical performance of the proposed algorithms. In order to apply the proposed algorithms to the real environment, we will implement a practical tracking application based on the proposed algorithm as future work. In the practical algorithm, we have to expand the tracking space from two to three dimensions and consider the limitations on research that the obstacles exist in the tracker’s path and target continues to move. We can consider reinforcement learning in how to optimize the tracking path to solve the limitations on research. We consider drones’ swarm-flight to implement a tracking system while using multiple receivers, and we can expect performance improvement through cooperation between drones through multi-agent reinforcement learning. Additionally, we remain future work to improve the accuracy of RSSI-based location measurement technology by combining it with video image-based location measurement technology.
